# Familial risks in and between stone diseases: sialolithiasis, urolithiasis and cholelithiasis in the population of Sweden

**DOI:** 10.1186/s12882-018-0945-y

**Published:** 2018-07-03

**Authors:** Kari Hemminki, Otto Hemminki, Anni I. M. Koskinen, Asta Försti, Kristina Sundquist, Jan Sundquist, Xinjun Li

**Affiliations:** 1 Division of Molecular Genetic Epidemiology, German Cancer Research Center (DKFZ), Im Neuenheimer Feld 580, D-69120 Heidelberg, Germany; 2 Center for Primary Health Care Research, Lund University, 205 02 Malmö, Sweden; 3 Department of Urology, Helsinki University Hospital, Helsinki, Finland; 4 Cancer Gene Therapy Group, Faculty of Medicine, University of Helsinki, Helsinki, Finland; 5 Department of Otorhinolaryngology, Helsinki University Hospital, Helsinki, Finland; 6 Department of Family Medicine and Community Health, Population Health Science and Policy, Icahn School of Medicine at Mount Sinai, New York, USA; 7 Department of Functional Pathology, Center for Community-based Healthcare Research and Education (CoHRE), School of Medicine, Shimane University, Matsue, Japan

**Keywords:** Salivary gland stones, Kidney stones, Ureter stones, Bladder stones, Heritability

## Abstract

**Background:**

According to the literature the three stone diseases, sialolithiasis (SL), urolithiasis (UL) and cholelithiasis (CL) share comorbidities. We assess familial and spouse risks between these stone disease and compare them to familial risks for concordant (same) stone disease.

**Methods:**

Study population including familiar relationships was obtained from the Swedish Multigeneration Register and stone disease patients were identified from nation-wide medical records. Standardized incidence ratios (SIRs) were calculated for 0–83 year old offspring when their first-degree relatives were diagnosed with stone disease and the rates were compared to individuals without a family history of stone disease. Numbers of offspring with SL were 7906, for UL they were 170,757 and for CL they were 204,369.

**Results:**

SIRs for concordant familial risks were 2.06 for SL, 1.94 for UL and 1.82 for CL. SIRs for SL and UL were slightly higher for women than for men. Familial risks between stone diseases were modest. The highest risk of 1.17 was for UL when family members were diagnosed with CL, or vice versa. The SIR for UL was 1.15 when family members were diagnosed with SL. Familial risks among spouses were increased only for UL-CL pairs (1.10).

**Conclusions:**

Familial risks for concordant SL were 2.06 and marginally lower for the other diseases. Familial risks between stone diseases were low but higher than risks between spouses. The data show that familial clustering is unique to each individual stone disease which would imply distinct disease mechanisms. The results cast doubt on the reported comorbidities between these diseases.

## Background

Sialolithiasis (SL) is a condition where a calcified mass (sialolith, salivary calculi or salivary stone) forms within the ducts of salivary glands [1, 2]. The most common location is the submandibular gland (Warton’s duct), while the parotid gland and particularly the sublingual gland are less frequently affected [3]. Reported incidence rates vary but in the Danish patient records the incidence was 7.3 per 100,000 person-years based on medically confirmed cases and doubled when unconfirmed cases were included [4]. Risk factors include infections, inflammation, diabetes and Sjögren syndrome [5]. Literature on possible familial risk or genetic predisposition in SL is practically non-existent [6]. Urolithiasis (UL) or urinary tract stone disease covering stones in the kidney (nephrolithiasis), ureter or bladder is a common disease with between 1 and 15% of people globally affected at some point in their life with the condition and the disease prevalence is increasing [7, 8]. Stone formation is due to a combination of genetic and environmental factors. Risk factors include high urine calcium levels, obesity, certain foods, some medications, calcium supplements, hyperparathyroidism, gout, diabetes, hypertension, not drinking enough fluids and family history [8, 9]. Cholelithiasis (CL) or gallstone disease is the most common of these three and some 10 to 20% of the populations in western counties develop gallstones and the condition is becoming more widespread due to the increasing prevalence of risk factors such as obesity and physical inactivity [10, 11]. Other risk factors are female sex, high age, pregnancy, certain ethnic background, family history and genetics [10, 12].

According to X-ray microanalysis stones in SL and UL share elemental composition and calcium salts are the main inorganic chemical constituents [1]. In contrast, gallstones are made of organic compounds, the most common ones are cholesterol stones and rarer ones are bilirubin stones [13]. Several epidemiological studies have observed comorbidities between these stone diseases. Case-control studies from Taiwan found significant increases for nephrolithiasis (odds ratio, OR 4.74) and CL (OR 2.20) in SL patients compared to controls [14, 15]. A similar study from the same source found a risk of renal stones (OR 1.68) in CL patients [16]. A prospective US study found reciprocal risks for renal stones and CL in the OR range of 1.2 to 1.6 in men and women [17]. Although the above and other studies make a case for true comorbidities among stone diseases, surveillance bias is a vicious intervening factor which is extremely difficult to avoid or correct for in diseases for which prior medical contacts have taken place [18].

Comorbidity for two diseases may be explained by shared environmental or genetic factors. We have a possibility to estimate the possible role of environmental factors among spouses, and we have an excellent possibility to address the possible role of genetic factors as we have data on all family relationships in Sweden [19]. In view of individual and medical importance of the three stone diseases it would be of outmost relevance to verify the possible comorbidities in a setting where surveillance bias might not operate. Sharing of familial risks between the stone disease would provide a mechanistic rationale that some genetic and/or shared environmental factors would also pose an individual risk for comorbidity. We use Swedish national health service and family records to assess familial risks for the three stone diseases. We assess spouse correlations between these diseases in order to quantify the possible risks through cohabitation.

## Methods

Family relationships were obtained from the Multigeneration Register, containing the Swedish population in families. ‘The offspring generation’ was born after 1931 and by year 2015 oldest offspring reached age 83 years; siblings could be defined only in the offspring generation. Stone diseases were identified using the nationwide Swedish Hospital Discharge Register (1987–2015) and the Outpatient Register (2001–2015). The first stone diagnosis was considered and a patient was only entered once, in order to avoid surveillance bias. Only 9.6% of SL patients were later diagnosed with UL or CL, and for UL and CL patients the proportions with multiple stone diseases were even lower. In a separate analysis, risks were calculated to offspring whose family members were diagnosed with a single stone disease or with multiple stone diseases. Information from the registers was linked at the individual level via the national 10-digit civic registration number. In the linked dataset, civic registration numbers were replaced with serial numbers to ensure anonymity. Revisions 9 (1987–1996) and 10 (1997-) of the International Classification of Diseases were used to identify SL, UL and CL patients.

Age-adjusted incidence rates for patients identified from the inpatient and outpatient registers were calculated per 100,000 person years of the population. For incidence trend plots only inpatient data were used as only these data were available from 1987 to 2015.

Familial risk was considered for offspring with a stone disease whose first-degree relatives (parent or siblings) were diagnosed with the same (concordant) or different (discordant) stone disease. Standardized incidence ratios (SIRs) were calculated as the ratio of observed to expected number of cases. The expected numbers were calculated for all individuals without a history of the defined stone disease, and the rates were standardized by 5-year-age, gender, period (5 years group), socioeconomic status and residential area. The expected rates were derived from the present dataset covering the Swedish population. The 95% confidence interval (95%CI) of the SIR was calculated assuming a Poisson distribution.

In order to assess environmental risk factors for familial stone disease, we determined SIRs for spouses. The period at risk for spouses was defined to start at the birth year of their first common child or at the first year that they were registered as living in the same address, whichever came first. The follow-up was terminated at stone disease diagnosis, death or when spouses no longer lived in the same address [20].

The study was approved by the Regional Ethical Review Board of Lund University (no. 2012/795).

## Results

In Table 1 we show characteristics of the populations used. The total Swedish population used for the study included 8.85 million individuals in the offspring generation and 8.09 million in the parental generation. Case numbers in the two generations were around 8000 for SL, around 200,000 for UL, and 200,000 for CL in offspring and over 300,000 for CL in parents. The median ages (i.e., age at first hospital contact) for all stone disease were in the 40s for offspring and in the 50s for parents. Incidence rates per 100,000 person years were in offspring and parents 4.1 and 3.6 for SL, 94.2 and 90.5 for UL, and 111.4 and 120.8 for CL.

**Table 1:** Population and number of cases of SL, UL and CL in offspring and parents

	SL		UL		CL	
	Offspring	Parents	Offspring	Parents	Offspring	Parents
Total population	8,850,394	8,090,648	8,850,394	8,090,648	8,850,394	8,090,648
Diagnosis of events						
No. of events	7906	8241	170,757	219,354	204,369	309,561
Mean age at diagnosis	43.8 ± 16.9	51.7 ± 16.8	47.8 ± 15.5	54.9 ± 16.8	47.1 ± 14.7	57.3 ± 18.2
Median age	45	52	49	55	47	58
Incidence rate per 100,000 person years^a^	4.1	3.6	94.2	90.5	111.4	120.8

^a^Age adjusted for European standardized population, *SL* sialolithiasis, *UL* urolithiasis, *CL* cholelithiasis

**Fig. 1:**
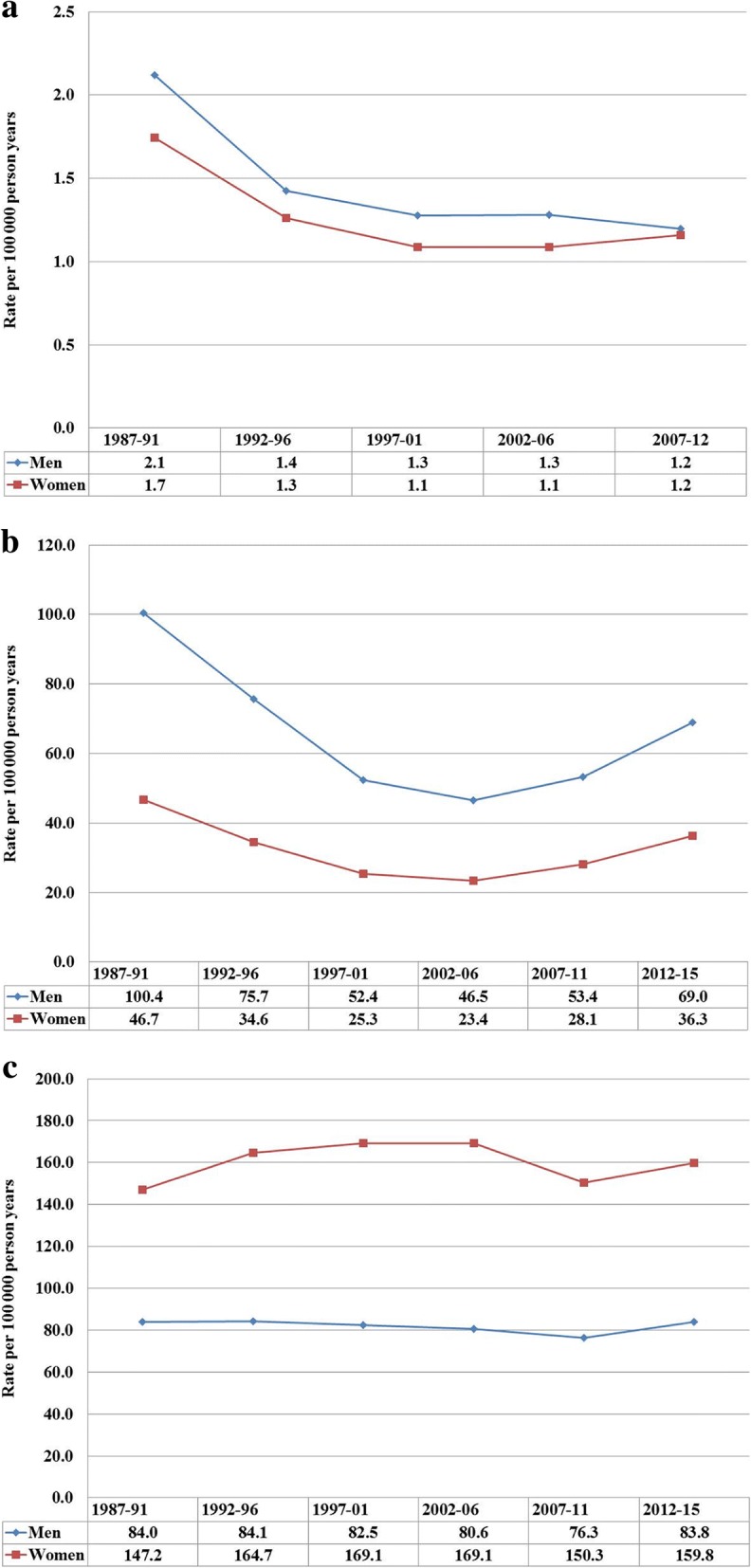
Age adjusted incidence rates (per 100,000 person years) for sialolithiasis (**a**), urolithiasis (**b**) and cholelithiasis (**c**) for Swedish inpatient during 1987 to 2015

Incidence trends for inpatients are shown in Fig. 1. Male rate were higher than female rates for SL and UL but for CL the opposite was the case. Note that the incidence in Fig. 1 is lower for SL and UL than the rates cited in Table 1 because for these diseases a large proportion of diagnoses originated from the outpatient register (see Methods).

Familial risks are shown in Table 2 for offspring when their parents were diagnosed with the same (concordant) stone disease. The overall familial risks were quite similar, 2.06 for SL, 1.94 for UL and 1.82 for CL. SIRs for SL and UL were slightly higher for women than for men.

**Table 2:** Concordant familial risk of SL, UL, and CL

	Men				Women				All			
Family history	O	SIR	95% CI		O	SIR	95% CI		O	SIR	95% CI	
SL	31	**1.95**	**1.32**	**2.77**	39	**2.15**	**1.53**	**2.94**	70	**2.06**	**1.60**	**2.60**
UL	19,234	**1.91**	**1.88**	**1.94**	11,221	**1.99**	**1.95**	**2.02**	30,455	**1.94**	**1.92**	**1.96**
CL	15,534	**1.82**	**1.79**	**1.85**	33,423	**1.81**	**1.79**	**1.83**	48,957	**1.82**	**1.80**	**1.83**

Bold type: 95% CI does not include 1.00

*O* observed number of cases, *SIR* standardized incidence ratio, *CI* confidence interval, *SL* sialolithiasis, *UL* urolithiasis, *CL* cholelithiasis

Discordant familial risks are shown in Table 3. The SIRs for both sexes were increased for all stone disease pairs with the exception of risk in SL when family members were diagnosed with UL. The highest risk of 1.17 was for UL when family members were diagnosed with CL, or vice versa. The SIR UL was 1.15 when family members were diagnosed with SL. For women 5 pairs were increased compared to only 2 pairs for men. The SIRs were equally high for men and women for UL-CL and CL-UL.

**Table 3:** Familial SIR of SL, UL, and CL

Risk in offspring	Family history	Men				Women				All			
		O	SIR	95% CI		O	SIR	95% CI		O	SIR	95% CI	
SL	UL	398	1.04	0.94	1.15	436	1.03	0.94	1.13	834	1.04	0.97	1.11
SL	CL	572	1.06	0.97	1.15	678	**1.14**	**1.05**	**1.23**	1250	**1.10**	**1.04**	**1.16**
UL	SL	494	1.09	0.99	1.19	340	**1.27**	**1.13**	**1.41**	834	**1.15**	**1.08**	**1.21**
UL	CL	5410	**1.17**	**1.14**	**1.21**	3316	**1.16**	**1.12**	**1.20**	8726	**1.17**	**1.14**	**1.19**
CL	SL	267	1.03	0.91	1.16	714	**1.18**	**1.09**	**1.27**	981	**1.14**	**1.07**	**1.21**
CL	UL	7379	**1.17**	**1.14**	**1.20**	16,445	**1.17**	**1.15**	**1.19**	23,824	**1.17**	**1.16**	**1.19**

Bold type: 95% CI does not include 1.00

*O* observed number of cases, *SIR* standardized incidence ratio, *CI* confidence interval; *SL* sialolithiasis, *UL* urolithiasis, *CL* cholelithiasis

Familial risks were calculated to offspring whose family members were diagnosed with a single stone disease or with multiple stone diseases (Table 4). As the family history of multiple stone diseases was rare, the SIRs for offspring with a family history of a single stone disease were essentially the same as those without specification of family history as shown in Table 2. SIRs for UL and CL were significantly lower for offspring with a family history of multiple stone diseases compared to those with a family history of a single stone disease.

**Table 4:** Concordant familial risk of SL, UL, and CL for individuals with a family member diagnosed with a single or multiple stone diseases

	Family history of stone diseases	O	SIR	95% CI	
SL	Only SL	63	**2.04**	**1.57**	**2.61**
	SL with others (UL or CL)	7	2.27	0.90	4.71
UL	Only UL	28,367	**1.94**	**1.92**	**1.97**
	UL with others (SL or CL)	2088	**1.72**	**1.65**	**1.80**
CL	Only CL	46,157	**1.82**	**1.81**	**1.84**
	CL with others (UL or SL)	2800	**1.59**	**1.53**	**1.65**

Bold type: 95% CI does not include 1.00

*O* observed number of cases, *SIR* standardized incidence ratio, *CI* confidence interval, *SL* sialolithiasis, *UL* urolithiasis, *CL* cholelithiasis

Familial risks for the pairs of stone diseases among spouses are shown in Table 5. The overall risk (1.10) was increased only for UL-CL pairs. The SIR for SL in husbands was 1.26 when wives were diagnosed with UL, and conversely it was 1.26 for in wives when husbands were diagnosed with SL.

**Table 5:** Risk of SL, UL, and CL in spouses

Risk in spouse	Spouse history	Husbands				Wives				All			
		O	SIR	95% CI		O	SIR	95% CI		O	SIR	95% CI	
SL	UL	100	**1.26**	**1.03**	**1.53**	200	1.01	0.88	1.16	300	1.08	0.96	1.21
SL	CL	37	0.82	0.58	1.13	69	1.20	0.93	1.51	106	1.03	0.84	1.25
UL	SL	200	1.00	0.86	1.15	100	**1.26**	**1.02**	**1.53**	300	1.07	0.95	1.20
UL	CL	9153	**1.10**	**1.07**	**1.12**	995	**1.12**	**1.05**	**1.19**	10,148	**1.10**	**1.08**	**1.12**
CL	SL	69	1.18	0.92	1.50	37	0.81	0.57	1.11	106	1.02	0.83	1.23
CL	UL	995	**1.12**	**1.05**	**1.20**	9153	**1.10**	**1.08**	**1.13**	10,148	**1.10**	**1.08**	**1.13**

Bold type: 95% confidence interval does not include 1.00

*O* observed number of cases, *SIR* standardized incidence ratio, *CI* confidence interval, *SL* sialolithiasis, *UL* urolithiasis, *CL* cholelithiasis

## Discussion

The available literature, cited in Introduction, describes comorbidities between the three stone diseases SL, UL and CL. However, as chronic comorbidities involve multiple medical examinations, risk estimation may be subject to surveillance bias [18]. Comorbidities may be caused by shared risk or susceptibility factors, and these can be also tested in the family setting. Spouses share environmental risk factors, which may be shared to a lesser extent also by first-degree family members, in addition to the shared genes. We thus wanted to approach the issue of comorbidity in the family setting which is not sensitive to surveillance bias to the same degree as comorbidities in the same individual. Another motivation of the present study was the lack of literature on familial SL. Even though SL is a rare disease we were able to identify over 8000 patients through the nation-wide registers. The reported chemical similarities between SL and kidney stone might implicate similar mechanisms of stone formation. The strengths of the study were its nation-wide scope, medical diagnostics of stone diseases and complete family records obtained from the Multigeneration Register essentially covering the Swedish population over a century [19]. The limitation was the lack of access to primary health care records.

The novel familial data on concordant SL showed a familial risk higher (2.06) than that for UL (1.94) or CL (1.82). Female risks where somewhat higher than male risks (2.15 vs. 1.95) which was also the case for UL but not for CL. Concordant familial risks for UL are well documented in the literature [9, 21–23]. Also many genes contributing to susceptibility to UL are known and these encode rare metabolic factors, including disturbances in calcium and oxalate balance [24–26]. However, for CL earlier studies were mainly based on case-control design which may have inaccuracies in reporting of cases in family members [27–29]. Several genes predisposing to CL have been identified and these include variants encoding apolipoproteins, lipid receptors and proteins involved in cholesterol metabolism [10, 11].

Correlation between spouses was marginal although between UL and CL the SIR of 1.10 was statistically significant. This could be explained by known risk factors, such as obesity and physical inactivity. Such low correlations suggest that surveillance bias may contribute to the reported comorbidities. Familial risks between stone diseases were also modest and they were not significant between SL and UL for which the stone composition is similar [1]. The SIR between UL and CL was 1.17, somewhat higher than the SIR between spouses which may imply minor sharing of genetic susceptibility. Similarly the SIRs between SL and CL (1.10 and 1.14) were at least statistically significant. Shared risk factors between these stone diseases could be inflammation and diabetes. Further evidence on unique familial clustering of each stone disease was shown for risks with family histories of a single stone disease which for UL and CL were higher than those with family histories of multiple stone diseases. Although explaining such small familial risks between stone diseases is speculative, they nevertheless provide another argument against shared disease mechanisms underlying the reported comorbidities.

The study covered a time span of 29 years and it is appropriate to consider to what extent diagnostic modalities and techniques might have changed over time. Diagnosis of SL is usually made by characteristic history and physical examination, confirmed by x-ray or by ultrasound. For UL x-ray examination was the standard diagnostic modality in the early period but it was replaced by computerized tomography (CT) by around year 2000 as the primary modality. For CL abdominal ultrasound and CT are the standard diagnostic tools. In Sweden, the number of CT instruments increased over the study period from 85 by 1989 to 200 by 2010 [30]. Undoubtedly also ultrasound technologies have improved over the study period. However, how improvements in diagnostic modalities might influence incident patient numbers cannot be predicted because higher precision may add new cases but remove false diagnoses. In Fig. 1 the incident rate for CL was stable over the period while for SL and UL the rates appeared to be declining. The interpretation of the rates is not simple because for SL and UL a large number of patients were seen in outpatient care and because we have no data on patients seen in primary care.

## Conclusions

The Swedish nation-wide data show high familial risks for each of the concordant stone diseases, and for SL the research findings were entirely novel. Risks between stone diseases among spouses were low and significant only for UL and CL. Familial risks between stone diseases were also low but higher than risks between spouses. Our findings show that familial clustering is unique to each stone disease and further indicate that the underlying disease mechanisms are distinct. The results cast doubt on excessive comorbidities between these three stone diseases.

## Abbreviations

CI: Confidence interval
CL: Cholelithiasis
OR: Odds ratio
SIR: Standardized incidence ratio
SL: Sialolithiasis
UL: Urolithiasis
